# Gut Microbiota Modulation of Dementia Related Complications

**DOI:** 10.14336/AD.2025.0108

**Published:** 2026-02-27

**Authors:** Xiaoqing Su, Yinghua Chen, Xingxing Yuan

**Affiliations:** ^1^First Clinical Medical College, Heilongjiang University of Chinese Medicine, Harbin 150040, China.; ^2^The Fifth Department of Acupuncture, First Affiliated Hospital of Heilongjiang University of Chinese Medicine, Harbin, Heilongjiang 150040, China.; ^3^Department of Internal medicine, Heilongjiang Academy of traditional Chinese Medicine, Harbin, Heilongjiang 150001, China.

**Keywords:** Gut microbiota, microbiota-gut-brain axis, neurodegenerative disorders, dementia, treatment strategies

## Abstract

Recent advances in microbial pathogen research have highlighted the potential of gut microbe-based microbial medicine. One of the most extensively studied biological pathways is the gut-brain axis, which has been shown to reverse neurological disorders. Evidence from animal-based studies of dysbiosis suggest complex behavioral changes, such as alterations in sociability and anxiety, can be modulated through gut microbiota. Specifically, mental disorders include major depression, bipolar disorder, and schizophrenia. Gastrointestinal diseases can be reversed by modulating gut microbiota. Dementia and its related mechanisms are also amenable to modulation of the gut microbiota. This review focuses on the role of gut microbiota in dementia by discussing the effects on depressive symptoms, cognitive function, mood, behavioral changes, chronic stress, and the prospects of the microbiota-gut-brain axis for dementia. Although animal models have revealed promising approaches for treating dementia through the modulation of the gut microbiota, it may be premature to incorporate these interventions into standard clinical practice. The heterogeneity of findings from clinical trials and randomized control trials has yet to convincingly demonstrate the efficacy of modulation in reversing dementia and its related complications.

## 1. Introduction

Globally, there is consensus on the need for effective interventions to address the unmet needs of caregivers, especially those providing care at home. Factors, such as living conditions, sex, and age, should be considered when addressing these unmet needs [1]. Monthly visits to long-term care facilities improve the quality of life (QoL) of patients with dementia and their caregivers. Government and individual interventions can lessen the trepidation of aging [2]. Effective dataset analysis from nursing homes and epidemiological studies on antipsychotic measures can enhance electronic medication administration records [3]. Despite improvements in training programs, health-related QoL and depressive symptoms have not significantly improved. Strategies to manage emotional stress and support patients with mild cognitive impairment (MCI) are crucial [4]. Better staff ratios and engagement of informal caregivers in smaller homes can optimize leisure time activities and treatment. Moreover, psychotropic drug use and higher agitation levels are associated with increased direct care time, leading to lower cognitive function [5].

There is a clear need for better interventions to improve staff knowledge in managing MCI [6]. Telerehabilitation delivery has shown potential clinical significance for Alzheimer's disease (AD) patients with dementia, with improvements in fluid cognition, cognitive function, and aerobic fitness [7]. At-home adherence to treatments delivered via telerehabilitation can be seen as a promising eHealth intervention to preserve behavioral and cognitive abilities. Greater adherence enhances skills and the perceived fit of demands, along with a good level of technological usability [8]. To assist older adults in successfully creating and managing meaningful daily occupations, it is essential to effectively address and manage behavioral symptoms while improving their performance and overall functional abilities [9]. Prescriptions of high-risk medications, such as strong anticholinergics, sedative-hypnotics, or antipsychotics, should not be targeted at patients using medication-specific educational mailings, as this may lead to inappropriate prescribing and increased risk of adverse outcomes [10]. Mindfulness-based cognitive therapy delivered online has proven effective in improving psychological outcomes and adaptive coping among family members of individuals with dementia, ensuring that the health conditions of their loved ones are maintained [11]. Although various training programs exist for caregivers, they should not rely solely on multiple-choice examinations but instead incorporate open discussions to evaluate caregivers’ reasoning and decision-making skills. Additionally, a basic understanding of human biology and diseases can instill confidence in untrained caregivers. Periodic refresher training on administering antipsychotic medications should align with evidence-based medicine [12, 13].

Personalized music therapy can reduce agitation and delusions among people living with dementia and decrease verbal agitation [14]. One way to foster friendlier interactions between people living with dementia and their caregivers is to work on speech impairments. The Lee Silverman Voice Treatment (LSVT LOUD^®^) has a greater accuracy for Parkinson's disease-related speech issues and improved communication [15]. Empowerment programs using the Montreal Cognitive Assessment and Mini-Mental State Examination scales have demonstrated improvements in memory, orientation, and attention among adults with MCI [16]. The Senior Companion Program Plus is a promising model for caregivers, enhancing their preparedness and knowledge about AD/dementia, which can reduce the burden of caregiving [17]. Based on the recommendations from the National Institute on Aging of the United States, pragmatic trials for people living with dementia should focus on psychosocial care, QoL, functional care and outcomes, and medical care and outcomes [18]. Functional medicine perspectives on dementia care, focusing on the gut microbiota via the gut-brain axis, have revolutionized the management of neurodegenerative diseases [19]. Dietary supplements, probiotics, and macromolecules demonstrate therapeutic potential in modulating the composition of the gut microbiota, thereby influencing metabolite profiles and conferring systemic health benefits across distant organ systems [20, 21]. While dementia care traditionally focuses on symptom management, emerging evidence from clinical trial and randomized clinical trial, and basic medical research using mouse models suggest the potential benefits of interventions targeting gut microbiota modulation. These modulations offer promising strategies for disease modification and extension of healthy longevity. This review discusses the most significant of these related studies and their potential to reverse dementia and its associated complications.

## 2. Focus on Gut Microbiota and Dementia

Chronic neurological diseases leading to dementia, such as multiple sclerosis (MS), AD, Parkinson's disease (PD), and stroke-induced systemic alterations, are increasingly being studied within the context of the microbiota-gut-brain axis [22, 23]. Alterations in serotonin levels within this axis provide another potential mechanism through which the gut microbiota influences dementia and its associated complications [24]. In addition, scutellarin, a purified flavonoid, modulates amyloid-beta (Aβ) assembly, thereby reversing cognitive impairment and neuroinflammation by regulating the gut microbiota and cAMP/protein kinase A (PKA)/cAMP response element binding protein (CREB)/histone deacetylase 3 (HDAC3) pathways in microglia [25]. The “leaky gut” theory posits that bacteria can enter the bloodstream through a compromised intestinal lining, leading to sepsis. gut microbiota-induced sepsis creates both neuro-inflammatory and pro-amyloidogenic conditions that contribute to plaque-related inflammation and exacerbate amyloid plaque deposition, which are well-documented events in the pathogenesis of dementia [26]. Studies on the microbiota-gut-brain axis suggest that gut microbiota metabolites are linked to neurodegenerative processes, such as vascular dementia, ischemic stroke, and AD [27]. Disruption of the circadian rhythm, metabolic alterations, and production of neurotoxic compounds by the gut microbiota may explain the association between sleep disorders and dementia [28]. Moreover, cognitive impairment and memory loss can result from a cascade of events initiated by gut microbiota dysbiosis, impaired intestinal integrity, immune dysregulation, increased brain barrier permeability, aberrant brain protein aggregation, and neuroinflammation [29]. For instance, vitamin B12 deficiency may impair memory function by altering the composition of the gut microbiota and hippocampal insulin signaling [30].

Periodontitis induces gut microbiota dysbiosis, disrupts the integrity of the intestinal epithelial barrier, and promotes the colonic inflammatory response. In an AD mice model, periodontitis exacerbated AD-related anxiety behaviors and cognitive deficits through mechanisms involving neuroinflammation, microglial morphological alterations, and elevated Aβ deposition in the brain [31]. Research on fatty acid profiles has demonstrated that reducing the omega-6/omega-3 ratio can modulate gut microbiota via probiotics, thereby influencing lipid profiles, inflammation, and insulin sensitivity [32]. Given that neurodegenerative disorders are susceptible to glycation, non-enzymatic modulation of gut microbiota glycation offers potential therapeutic avenues for dementia [33]. Modulation of the gut microbiota can influence inflammation, oxidoreductase status, endocrine and immune systems, and the stress-dysbiosis axis, providing potential strategies to intervene in the neuropathological cascade of dementia [34]. Electroacupuncture enhances the intestinal barrier by upregulating beneficial bacteria in the gut microbiota, which in turn strengthens the blood-brain barrier, attenuates hippocampal inflammatory responses, and improving postoperative cognitive dysfunction [35].

### 2.1 Influence of the Gut Microbiota on Depressive Symptoms

Prolonged stressful conditions and adverse life events are well-established contributors to depression. Given the multifactorial nature of depression, understanding the interplay between stress and associated physiological changes, including dysbiosis, is pivotal for effective management [36, 37]. Studies comparing depression in individuals to healthy controls have revealed disparities in both α-diversity (number of distinct species present in the given sample) and β-diversity (degree of community differentiation) of the gut microbiota [38]. In addition to dysbiosis, neurotransmitter imbalances [39], hypothalamic-pituitary-adrenal (HPA) axis activation, pathogen-induced inflammation, and oxidative stress are thought to be influenced by the microbiota-gut-brain axis [40]. These processes may subsequently modify the brain-derived neurotrophic factor (BDNF) levels. Decreased BDNF levels have been associated with depressive conditions [41]. Subclinical inflammation and HPA axis are common in patients with mental disorders. Cytokines, microbial antigens, and prostaglandins cross the blood-brain barrier and activate the HPA axis. Short-chain fatty acids (SCFAs) play a key role in counteracting the effects of excessive glucocorticoid production [42].

Major depressive disorder (MDD) is associated with chronic pain, poor treatment adherence, and metabolic disturbances [43]. Treatment resistance, especially in MDD, remains a significant challenge for current antidepressants. The therapeutic latency can extend from several weeks to months, and gastrointestinal diseases are prevalent in patients with bipolar disorder [44]. Patients experiencing the depressive phase of bipolar disorder require rapid and effective treatment with antidepressants [45]. Psychiatric symptoms may be improved by a gluten-free diet combined with probiotic supplementation, which can inhibit the immune-inflammatory cascade found in MDD [46]. Preventive measures, such as psychobiotics and fecal microbiota transplantation (FMT), aim to enhance the microbiota-gut-brain axis [47].

Pathophysiology of depression is associated with gut microbiota metabolism. However, a holistic approach is required to understand the neuroactive potential of microbial-derived molecules [48]. Depression that develops later in life is often accompanied by comparative impairment that persists despite treatment [49]. Depression affects structural and functional brain plasticity, particularly in the subcortical limbic regions and frontal lobe[50]. Localization of cerebrovascular lesions, sex-specific differences, the tau hypothesis, neuroinflammatory processes, Aβ accumulation, and BDNF alterations are all relevant factors. Recent data have highlighted the role of epigenetics and the microbiota-gut-brain axis [51]. Depression is associated with gut microbiota alterations at the phylum and genus levels, as well as the fecal metabolic phenotype [52]. Notably, depressive symptoms corelate with alterations in the gut microbiota, particularly in members of the genus *Paraprevotella* [53]. Irritable bowel syndrome, characterized by the correlation of microbial composition and psychological distress, shows a significant association between β-diversity and depression, suggesting a potential biomarker for psychological distress [54]. Patients with newly diagnosed bipolar disorder exhibit increased levels of *Flavonifractor*, which correlate with smoking habits [55].

Elevated gut permeability contributes to systemic pro-inflammatory states by allowing bacterial translocation and immune activation, which have been implicated in neuropsychiatric disorders [56]. In antenatal populations, supplementation with *Lactobacillus rhamnosus* HN001 significantly lowers depression scores and reduces postpartum anxiety and depression [57]. Similarly, fructooligosaccharides possess antidepressant properties by modulating the gut microbiota, potentially by promoting the abundance of the bacterial phylum *Cyanobacteria* [58]. Importantly, reduced levels of *Lactobacillus* and *Bifidobacterium* are typically observed in patients with MDD, offering new insights into the prebiotic- and probiotic-mediated pathophysiology of MDD [59]. Furthermore, the gut microbiota composition critically influences the clinical outcomes of patients with severe mental illnesses. For instance, gut microbiota α-diversity observed during early hospitalization phases correlates with heightened inflammatory markers, suggesting a mechanistic link between microbial diversity deficits and the inflammatory underpinnings of psychiatric pathophysiology [60] (Table 1).

**Table 1:** Effects of gut microbiota on depressive symptoms.

Aim of study	Main Findings	Conclusions	Ref.
**■ Impacts of “leaky gut” on depression and marital distress**	■ Increased lipopolysaccharide binding protein (LBP) and LBP/sCD14 ratio were associated with higher levels of C-reactive protein	■ Increased gut microbiota permeability may exacerbate marriage distress and mood disorders	[56]
**■ Cognitive functions in** **patients with major depressive disorder (MDD)**	■ Improved cognitive performance ■ Decreased kynurenine concentration	■ Improvement of cognitive functions	[59]
**■ Impacts of almond-based low carbohydrate diet on glycometabolism and depression**	■ Improved depression and level of hemoglobin A1C ■ Increased abundance of *Roseburia*, *Ruminococcus*, and *Eubacterium*	■ Beneficial effect on glycometabolism and depression	[61]
**■ Gut microbiota-parasite interactions**	■ Depression associated with gut microbiota perturbation	■ *Ascaris lumbricoides* can induce depressive symptoms	[62]
**■ Probiotic supplementation on depressive symptoms**	■ Reduced cognitive reactivity ■ No significant alteration in gut microbiota composition ■ Correlation between *Ruminococcus gnavus* and one depression metric	■ Probiotics can influence psychological variables associated with depression	[63]
**■ Impact of nutritional education on psychological factors in obese women**	■ Increased consumption of milk products ■ Decreased depression scale scores	■ Nutritional education targeting gut microbiota composition can improve psychological outcomes	[64]
**■ Probiotic treatment in depressed individuals**	■ Increased abundance of *Ruminococcus gauvreauii* and *Coprococcus 3* ■ Enhanced β-diversity ■ Modulation of inflammation-regulatory and metabolic pathways	■ Probiotic has limited clinical significance in treating depression	[65]
**■ Probiotic and prebiotic interventions in patients with MDD**	■ Decreased Beck Depression Inventory scores and kynurenine/tryptophan ratio in probiotic group ■ No significant effect of prebiotics	■ Probiotics, but not prebiotics, show promise in improving mood in MDD patients	[66]
**■ Impacts of probiotics on cognition and mood**	■ Shift in *Eubacterium* and *Clostridiales* populations	■ Probiotics promote mental flexibility and alleviate stress	[67]
**■ Impact of probiotic supplementation in MDD patients on gluten-free diets**	■ Digested gluten forms peptides ■ Different immunogenic capacities	■ Probiotics reduce immune-inflammatory cascades in MDD patients on gluten-free diets	[68]
**■ Chaihu-Shugan-San (CSS) on functional dyspepsia**	■ Improved symptoms of functional dyspepsia ■ Shift in bacterial populations	■ CSS alleviates functional dyspepsia-related anxiety and depression	[69]
**■ Impact of ketogenic diet on MDD symptoms**	■ Improved MDD symptoms	■ Ketogenic diet may have therapeutic potential for MDD	[70]
**■ Impact of diverse probiotic fiber on metabolic syndrome**	■ Reduced high sensitivity C-reactive protein ■ Increased abundance of *Bifidobacterium* and *Parabacteroides*	■ Diverse prebiotic cocktails can relief mood, stress, and anxiety	[71]
**■ Impact of high doses of dietary micronutrients on antenatal depression**	■ Reduced α- and β-diversity ■ Decreased *Coprococcus* with micronutrient administration ■ Increased *Coprococcus* with α-diversity in antenatal depression	■ Micronutrient supplementations support stable gut microbiome diversity during pregnancy	[72]
**■ Impact of probiotics on immunometabolic depression**	■ Improved aspartate transferase to platelet ratio index ■ Higher baseline levels of intestinal permeability markers	■ Probiotics may mitigate immunometabolic forms of depression	[73]

### 2.2 Impact of Gut Microbiota on Cognitive Function

Recent research has increasingly clarified the relationship between gut microbiota and cognitive function. The combined administration of selective serotonin reuptake inhibitors and *Lactobacillus plantarum* 299v enhances cognitive performance by reducing kynurenine concentrations in patients with MDD [74]. Activation of the mediates gut-brain communication and behavioral adaptations are mediated through vagus nerve activation, which upregulates pro-inflammatory cytokines, including tumor necrosis factor-alpha (TNF-α) and interleukin-6 (IL-6), and leads to the accumulation of carbonylated proteins, reflecting systemic oxidative stress [75]. The gut microbiota actively regulates neurotransmitters and SCFAs production. For example, *Allobaculum* is positively correlates with the production of acetic acid and 5-hydroxytryptamine (5-HT) [76]. Data from donor and recipient mouse models further establish a strong association between the gut microbiota and depression, suggesting that targeted manipulation of the gut microbiota composition may serve as a preventive strategy against depressive disorders [77]. Schisandrin, produced in response to lipopolysaccharide, reduces the levels of pro-inflammatory factors and the expressions of Toll-like receptor 4 and nuclear factor-kappa B in the brain, thereby mitigating neuroinflammation [78]. Deficiencies in *Firmicutes*, a key bacterial phylum, result in reduced SCFA production, contributing to the inflammatory cascade observed in patients [79].

Diet-induced depression is associated with specific gut microbiota-related metabolites including phosphorylation of transient receptor potential vanilloid 1 (TRPV1), altered BDNF/tropomyosin receptor kinase B signaling, and disrupted neuronal firing in the hippocampus [80]. Probiotics confer beneficial changes in the gut microbiota composition, subsequently attenuating the release of pro-inflammatory cytokines such as TNF-α, interferon-gamma (IFN-γ), and IL-6 upon mitogen stimulation [81]. Moreover, *Bifidobacterium breve* CCFM1025 exhibits antidepressant-like effects by alleviating hyperactive HPA axis responses, potentially through the regulation of glucocorticoid receptor expression (*Nr3c1*) [82]. N-(2-(7-Methoxy-3, 4-dihydroisoquinolin-1-yl) ethyl) acetamide hydrochloride can attenuate depression-like behaviors by modulating gut microbiota-associated pro-inflammatory cytokines. This effect involves the microbiota-gut-brain axis, which regulates neuro-inflammatory markers [83]. Furthermore, the traditional Chinese herbal formula Kai-Xin-San (KXS) exerts antidepressant-like effects by regulating the microbiota-gut-brain axis, highlighting the therapeutic potential of gut microbiota-targeted interventions [84] (Table 2)

**Table 2:** Effects of gut microbiota on cognitive function.

Aim of study	Finding	Conclusion	Ref.
**■ Impact of acarbose and glipizide on type 2 diabetes mellitus patients**	■ Increased abundance of *Lactobacillus* and *Bifidobacterium* ■ Depletion of *Bacteroides*	■ The increased and decreased abundance may provide a tool for predicting antidiabetic metabolic benefits	[85]
**■ FMT in metabolically uncompromised obese patients**	■ Shifts in microbiome composition ■ Decreased taurocholic acid	■ FMT is safe but does not significantly reduce body mass index in obese patients	[86]
**■ Cognitive impairment**	■ Improvements in cognitive functions ■ Enhanced telomere integrity ■ Increased plasma BDNF	■ Cognitive capacity influences gut microbiota profiles	[87]
**■ Impact of dietary fiber polydextrose on cognitive performance**	■ Decreased errors in the Intra-Extra Dimensional Set Shift task ■ Higher responses and rejections in the Rapid Visual Information Processing task	■ Polydextrose may modulate behavioral responses via gut-brain communication	[88]
**■ Impact of prebiotic inulin on psychological parameters**	■ Increased interleukin-8 (IL-8) ■ Improved insulin resistance ■ Reduced adiposity and metabolic/inflammatory profiles	■ Inulin intake can improve mood	[89]
**■ Impact of daily legume consumption on fecal microbiota**	■ No significant alterations in gut microbiota across age groups	■ Legume consumption does not significantly affect gut microbiota communities	[90]
**■ Impact of probiotics on anxiety**	■ Improvements in panic and anxiety ■ Negative affecting worry ■ Enhanced negative mood regulation	■ Probiotics efficacy varies depending on species count	[91]
**■ Impacts of branched chain amino acids and resistance exercise on cognition**	■ Changes in gut microbiota with increased *Bifidobacterium* ■ No significant difference in chair-rise performance	■ Prebiotics may improve cognition function, but not physical performance	[92]
**■ Impacts of dance classes on motor and cognitive functions in MS**	■ Favorable shifts in gut microbiota communities ■ Increased abundance of *Blautia stercoris* ■ Decreased abundance of *Ruminococcus torques*	■ Dance classes improve motor and cognitive functions in MS patients	[93]
**■ Impact of vitamin D supplementation on psychiatric symptoms in schizophrenic patients**	■ Increased Montreal Cognitive Assessment scores ■ No significant difference in Positive and Negative Syndrome Scale scores	■ Co-administration of probiotics and vitamin D may improve cognitive function	[94]
**■ Prospective association of antibiotics with cognitive aging**	■ No increased risk of dementia ■ No association of antibiotic use and dementia risk factors	■ No evidence linking gut microbiota and antibiotic-associated dementia risk	[95]
**■ Metformin on type 2 diabetes mellitus-associated cognition**	■ Positive association with Proteobacteria (*Escherichia coli*) and Verrucomicrobia (*Akkermansia muciniphila*) ■ Negative association with Firmicutes phylum, *Romboutsia ilealis*, and *Romboutsia timonens*is ■ *Akkermansia muciniphila* / *Romboutsia ilealis* ratio associated with cognition	■ Metformin-induced gut microbiota and microbial-host-derived co-metabolites alterations may influence cognition	[96]

### 2.3 Influences of Gut Microbiota on Mood and Behavioral Changes

The gut microbiota plays a crucial role in modulating mood and behavioral changes across different age groups. In toddlers, gut microbiota diversity is strongly associated with temperament, suggesting an early link between microbial composition and emotional development [97]. Among college students, anxiety-related conditions, such as panic, neurophysiological anxiety, and worry, are linked to gut microbiota alterations, highlighting the role of the microbiota-gut-brain axis in mental health [91]. Pharmacological interventions further underscore the influence of gut microbiota on brain function. For instance, rifaximin, a non-absorbable antibiotic, can modulate gut microbiota composition, thereby improving brain function [98]. Probiotics confer beneficial effects on the brain through activation patterns during decision-making tasks [99]. Metabolically, key pathways that include the starch, pentose phosphate, and sucrose metabolism pathways are implicated in gut microbiota-induced depression, providing mechanistic insights into how microbial activity influences mental health [100]. Additionally, the absence of *Clostridia* and reduced levels of *Bacteroides* have been identified as biomarkers of depression and anxiety, respectively, indicating that gut microbiota alterations correlate with comorbid clinical presentations [101].

Interventions targeting the gut microbiota composition have demonstrated therapeutic potential. For example, *Lactobacillus fermentum* NS9 normalizes gut microbiota composition and alleviates ampicillin-induced elevations in mineralocorticoid and N-methyl-D-aspartate receptor levels, thus benefiting memory retention [102]. Furthermore, examination of neuronal circuits has revealed an association between gut microbiota colonization and signaling molecules, providing a neurobiological basis for the impact of the gut microbiota on behavior [103]. The gut microbiota also offers partial protection against mania-like behavior, as demonstrated by the administration of GBR12909 [104]. Additionally, gut microbiota modulation activates the HPA axis via neuroimmune responses, elevating corticosterone levels and influencing stress-related behaviors [105]. Antibiotic-induced gut microbiota changes, such as those caused by ampicillin and cefoperazone, result in behavioral alterations, further reinforcing the microbiota-gut-brain connection [106]. Finally, elevated BDNF levels and increased exploratory behavior highlight the gut microbiota’s role in shaping brain biochemistry and behavior [107] (Table 3).

### 2.4 Impacts of Gut Microbiota on Chronic Stress

The gut microbiota plays a critical role in mediating chronic stress through complex interactions with the host physiological and neurochemical systems. For instance, *Bifidobacterium pseudocatenulatum* CECT 7765 downregulates maternal separation-induced intestinal inflammation during infancy, subsequently modulating the HPA axis and contributing to chronic stress development [118]. Dietary factors also influence gut microbiota-host interactions, as evidenced by the alteration of intestinal tight junction proteins by high-fat, high-fructose diets, which exacerbate metabolic syndrome-related conditions and adversely affect mouse behavior [119].

**Table 3:** Effects of gut microbiota on mood and behavioral changes.

Aim of study	Finding	Conclusion	Ref.
**■ Impacts of four weeks of probiotic administration on behavior, brain function, and gut microbiota composition in healthy volunteers**	■ Changes in brain activation patterns during emotional memory and decision-making tasks ■ Subtle shift in gut microbiota profile	■ Microbiome composition correlates with self-reported behavioral measures and memory performance	[99]
**■ Microbiota transfer therapy in autism spectrum disorder patients**	■ Improved behavioral symptoms ■ Beneficial changes in gut microbiota	■ A promising approach to alter gut microbiota and virome in autism spectrum disorder patients	[108]
**■ Impact of inulin-type fructans-rich vegetables on gut microbiota**	■ Increased abundance of *Bifidobacterium* ■ Decreased levels of unclassified *Clostridiales* and *Oxalobacteraceae*	■ Inulin-type fructans-rich vegetables may improve dietary fiber tolerance and food-related behavior	[109]
**■ Fermented milk product with probiotic in healthy women**	■ Reduced task-related response in affective, viscerosensory, and somatosensory cortices	■ Fermented milk product with probiotic alters midbrain connectivity, potentially explaining observed differences in brain activity during tasks	[110]
**■ Ten-week nature-related "Play & Grow" program**	■ Regulation of *Roseburia* abundance and fecal-serotonin levels ■ Reduced perceived stress	■ Nature-related activities connect children to nature and reduce stress	[111]
**■ Evaluation of a new psychobiotic formulation**	■ Significant reduction in Hamilton Anxiety Rating Scale scores	■ Psychobiotic represent a viable approach to address obesity and behavior disorders	[112]
**■ Impact of feather intake on chicken gut microbiota**	■ Lowered bacterial diversity and richness and shannon index ■ Changes in microbial metabolites, especially in the cecum	■ Feather intake induces gut microbiota adaptation in chickens	[113]
**■ Native inulin as a prebiotic** **in obese patients**	■ Lowered energy intake, body mass index, systolic blood pressure, and serum gamma-glutamyl transferase ■ Additionally, decreases in diastolic blood pressure, AST and insulinemia	■ Inulin-enriched diets promote weight loss in obese patients	[114]
**■ Probiotic interventions and host health via enhanced antioxidant activity**	■ Improved serum concentration of glycemic markers ■ Promoting better overall health	■ Probiotics may help regulate blood sugar, glycosylated hemoglobin levels and reduce weight	[115]
**■ Impact of Mediterranean-style dietary pattern on mood**	■ Expected outcomes include cognitive function, sleep quality, and brain perfusion	■ A linear mixed model will assess changes in cognition over time	[116]
**■ Impacts of *Bifidobacterium breve* 207-1 on behavioral lifestyle**	■ No significant change in mood scale ■ Decreased Pittsburgh Sleep Quality Index scores, indicating improved sleep quality ■ Slight increase in exercise consumption	■ Supplementation with *B. breve* 207-1 enhances daily lifestyle behaviors of healthy adults	[117]

Mechanistically, inflammasomes mediated by the microbiota-gut-brain axis has been implicated in anxiety-like behaviors, with caspase-1 inhibition serving as a key regulatory point in this pathway [120]. Post-traumatic stress disorder, behavioral changes, and neurotransmitter levels are further mediated by the gut microbiota composition. For example, a significant reduction in brain 5-HT levels has been linked to gut microbiota alterations, with the abundance of *Bacteroidaceae* showing a strong positive correlation with 5-HT concentrations, whereas an increased abundance of the RF32 class of the *Proteobacteria* exhibits an inverse relationship. Additionally, specific phyla, such as *Firmicutes*, *Bacteroidetes*, *Cyanobacteria*, and *Proteobacteria* show distinct linkages to fear- and anxiety-related behavioral phenotypes, which are mechanistically tied to their capacity to suppress 5-HT biosynthesis [121].

**Figure 1.**
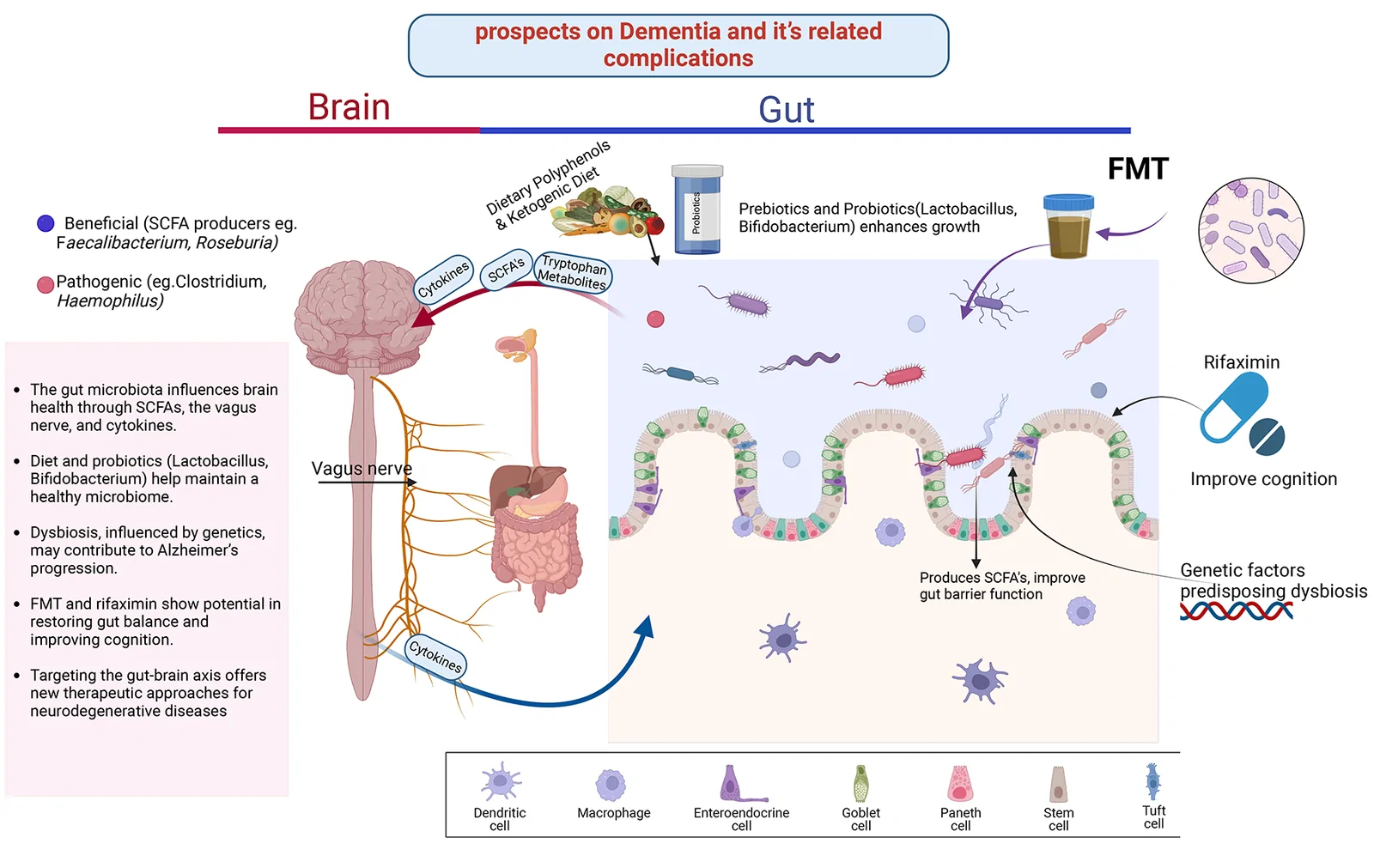
The roles of gut microbiota on depression, stress, moods and behavioral changes.

Notably, sex-specific neurochemical effects were observed in a germ-free animal model, with significant increases in hippocampal 5-HT and its main metabolite, 5-hydroxyindoleacetic acid, in male germ-free animals, in stark contrast to the conserved immune and neuroendocrine responses observed in both sexes [122]. Central nervous system neurotransmission exhibits strong transgenic dependence; despite subsequent microbial colonization, these neurochemical features persist. Emerging therapeutic strategies targeting the gut microbiota have shown promise in alleviating stress-related behaviors. For example, treatment with raw and wine-processed *Schisandra chinensis* improves anxiety- and depression-like behaviors. This effect is mediated by the modulation of gut microbiota derivatives, which activate the GPR81-mediated lipid metabolism pathway, providing a mechanistic link between gut microbiota composition and behavioral outcomes[123] (Fig. 1).

## 3. Prospects of the Microbiota-Gut-Brain Axis for Dementia

Therapeutic interventions aimed at restoring gut dysbiosis include dietary modification, pre- and probiotics, and FMT [124, 125]. Eating disorders are often associated with psychiatric comorbidities, and patients with these conditions exhibit altered microbiota in the upper and lower gastrointestinal tracts [126]. Reversing these microbial imbalances throughout the gastrointestinal tract may promote good eating habits and alleviate psychiatric comorbidities. Specifically, FMT has been implicated in the treatment of schizophrenia [127]. However, to avoid consequences similar to those of blood group incompatibility, the safety and efficacy profiles of this treatment option require large longitudinal studies. Probiotics enhance the gut microbiota by facilitating gene and metabolite exchange among commensal bacteria, thereby modulating immune and epithelial cell activity [128]. Synbiotics also hold promise for the treatment of neuropsychiatric disorders; however, their underlying mechanisms require deeper investigation [129].

Concerning the gut microbiota, nutrition plays a crucial role in brain health, particularly in the context of dementia, with minimal risk or side effects [130]. Dietary polyphenols have been studied for their role in cognitive resilience in neuropsychiatric disorders. Although the interaction between the gut microbiota and dietary polyphenols is not fully understood, polyphenols have demonstrated therapeutic efficacy in the treatment of depression and mood disorders [131]. The ketogenic diet, an alternative treatment for drug-resistant epilepsy, is influenced by the gut microbiota [132]. Additionally, targeting attention deficit hyperactivity disorder via gut microbiota modulation requires a comprehensive molecular approach [133]. It has been hypothesized that dementia can be reversed by altering the metabolic output of gut microbiota, which is influenced by glutamate [134]. However, N-methyl-D-aspartate glutamate receptor-enhancing agents have yet to be explored for the treatment of neurodegenerative dementias [135].

Rifaximin is a gut-specific antibiotic that has shown promise for improving cognition in patients with minimal hepatic encephalopathy. Studies have demonstrated improvements in cognition, including working memory and inhibitory control, as well as enhanced frontoparietal and subcortical activation and connectivity following rifaximin therapy [136]. Bacterial lipopolysaccharide translocation, which can occur due to increased gut permeability, may contribute to mood disorders and promote a pro-inflammatory environment, highlighting the “gut feeling” phenomenon [137]. Furthermore, rifaximin modulates social stress and brain function, similar to probiotics [98].

**Figure 2.**
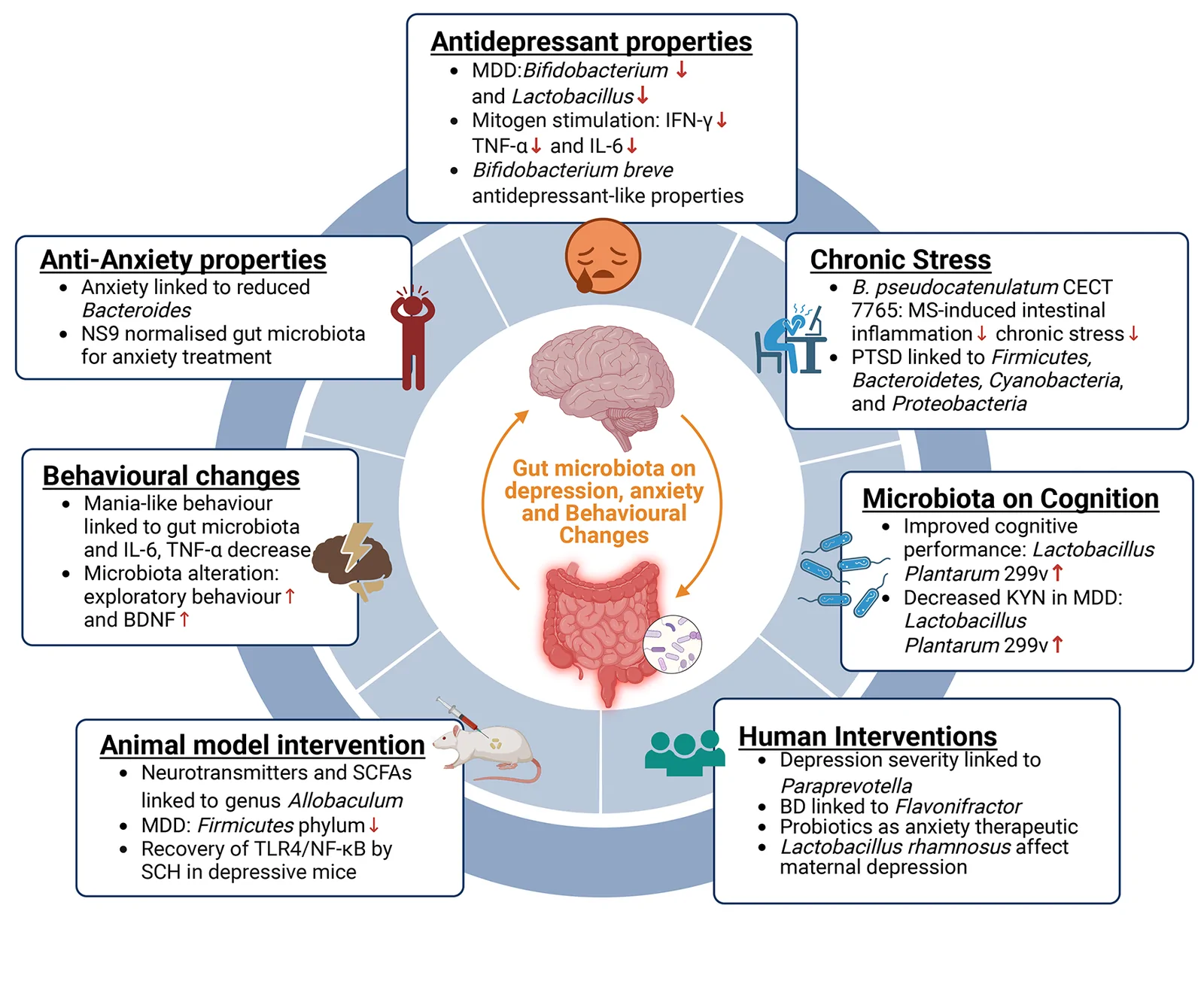
Prospects in dementia and its related complications.

Gut microbial metabolites influence depressive behaviors through various mechanisms, including direct stimulation of central receptors, peripheral activation of endocrine and immune mediators, neural pathways, and epigenetic regulation via DNA methylation and histone acetylation [138]. Such multifactorial interactions underscore the role of the gut-brain axis in mood disorders. Emerging evidence suggests that gut microbiota dysbiosis may partially explain the etiology of Pediatric Autoimmune Neuropsychiatric Disorders Associated with Streptococcal Infections (PANDAS) [139]. Genetic variations in key immune regulators, specifically polymorphisms in the mannose-binding lectin and TNF-α genes—have been implicated in PANDAS pathophysiology and could serve as diagnostic biomarkers. Maternal transfer of IgA during breastfeeding supports neonatal gut microbiota maturation, which protects against immune system development [140]. The correlation between early life breastfeeding and the risk of depression and mood changes in later life merits further investigation. The relationship between psychosocial stress, such as traumatic life events and HPA axis dysregulation remains an unclear scholarly project [141]. Oxygen-ozone (O_2_-O_3_) therapy is a treatment option for cognitive frailty with few or no side effects [142].

Tryptophan is an essential amino acid and a precursor of several molecules involved in the host-microbiota interface and is fundamental in modulating the microbiota-gut-brain axis [143]. As a precursor of 5-HT, the metabolism of tryptophan directly affects the central and peripheral availability of 5-HT. Impaired 5-HT function affects individuals across all life stages, from neonates to the elderly, and is associated with various psychiatric conditions. Since the modulation of tryptophan metabolism by the gut microbiota has been established in preclinical studies and supported by clinical evidence, new therapeutic strategies are possible [144]. Although mental well-being and its association with probiotic-mediated gut microbiota require further study, salivary metabolites may play a role in oral physiology and food behavior [145]. The peripheral aspects of the microbiota-gut-brain axis remain understudied and require extensive research. Photobiomodulation therapy, although in its early stages, shows promise in managing psychiatric disorders [146]. Genetic associations with the gut microbiota have been identified in conditions such as autism spectrum disorder and MDD, with specific variants, such as rs9401458, rs9401452, and rs75036654, playing significant roles [147] (Fig. 2).

## 4. Conclusion

Complex behavioral changes, such as alterations in sociability and anxiety, have extensively been studies in animal-based models of dysbiosis and mental disorders, including major depression, bipolar disorder, and schizophrenia. In humans, it has been linked to increased negative mood as well as reductions in anxiety, panic, neurophysiological stress responses, suggesting that probiotics on mental health issues may directly modulate the gut microbiota to influence brain physiology and functions. Additionally, stimulation of immune mediators, peripheral stimulation, activation of the central receptors of the endocrine system, neural and DNA methylation, and epigenetic regulation of histone acetylation are known biological phenomena. Prospects are based on multiple approaches. Diet-induced microbiota modulation is known to be associated with the reversal of depression and mood. However, in addition to elucidating metabolic output at the molecular level, HPA axis activation is imperative. The use of probiotics, prebiotics, and synbiotics may lead to a paradigm shift in the treatment of depression and mood disorders.
